# miR-199a-5p Plays a Pivotal Role on Wound Healing via Suppressing *VEGFA* and *ROCK1* in Diabetic Ulcer Foot

**DOI:** 10.1155/2022/4791059

**Published:** 2022-04-07

**Authors:** Hongshu Wang, Xianyi Wang, Xiaomin Liu, Jinbao Zhou, Qianqian Yang, Binshu Chai, Yimin Chai, Zhongliang Ma, Shengdi Lu

**Affiliations:** ^1^Department of Orthopedic Surgery, Shanghai Jiao Tong University Affiliated Sixth People's Hospital, 600 Yishan Road, Xuhui District, Shanghai 200233, China; ^2^Lab for Noncoding RNA & Cancer, School of Life Sciences, Shanghai University, Shanghai 200444, China; ^3^Shanghai New Tobacco Product Research Institute, Shanghai 201315, China

## Abstract

Diabetes mellitus (DM) is a growing health problem. As a common complication of DM, diabetic foot ulcer (DFU) results in delayed wound healing and is a leading cause of nontraumatic amputation. miR-199a-5p, a short noncoding RNA, had abnormal expression in DFU wound tissues. The expression of miR-199a-5p was significantly increased in DFU wound tissues, skin tissues of diabetic rats, and high glucose-induced cells. Vascular endothelial growth factor A (VEGFA) and Rho-associated kinase 1 (ROCK1) are directly targets of miR-199a-5p. Inhibiting the expression of miR-199a-5p alleviated the inhibition of VEGFA and ROCK1, thereby rescued impaired proliferation and migration of HG-induced cells, and restored the normal function of the cells to some extent. In diabetic rats, inhibition of miR-199a-5p significantly increased the expression of VEGFA and ROCK1, significantly promoted wound healing, and rescued impaired wound healing. miR-199a-5p and its targets showed therapeutic effect on diabetic wounds.

## 1. Introduction

Diabetic foot ulcer (DFU) is one of the major complications of diabetes [1]. The formation of DFU is closely related to the metabolic disorder of diabetes. It includes infections, anabrosis, and the damage to foot tissue, which has affected nearly 6% of the patients with diabetes [2]. DFU will affect 15% of diabetic patients and have risk of amputation [3]. The 5-year mortality of patients with this disease is about 2.5 times higher than people with no DFU [4, 5]. As a disabling disease, research on its etiology and treatment is of great significance.

VEGFA is a class of vital growth factors that involved in the diabetic wound healing. As a stimulator, it would bind with specific receptors and cause a series of intracellular signal transduction reactions [6]. The signaling cascade mediated by VEGFA can regulate the proliferation, migration, survival of vascular endothelial cells, and control angiogenesis [7, 8]. ROCK1 signaling modulates cell adhesion and cytoskeletal stretching upon cell migration, which has significant implications for cancer metastasis [9, 10].

MicroRNA (miRNA) is a kind of noncoding RNAs, about 22 nucleotides (nts) in length. miRNAs have been reported that they were critical regulatory molecule in DFU [11, 12]. miR-199a plays an important role in tumorigenesis, such as lung cancer [13, 14], hepatocarcinoma [15, 16], and ovarian cancer [17, 18], but roles in diabetes or DFU are unclear. There were limited studies that clarified the important roles of miR-199a-5p in the wound healing progress of diabetic ulcer foot. Wu et al. found that high glucose increased miR-199a-5p expression and induced the inflammatory reaction in rat mesangial cells [19]. Although the function of miR-199a-5p to inhibit the cell proliferation and migration in various types of tumors has been verified [13, 20], whether it is involved in the wound healing phase of diabetic ulcer foot is still unknown. Therefore, in this study, we have a hypothesis that miR-199a-5p plays a critical role via VEGFA and ROCK1 in the wound healing progress of DFU. Our research will give a new potential drug target for diabetic wound healing.

## 2. Materials and Methods

### 2.1. Clinical Tissue Samples and Cell Lines

DFU tissues were collected from the Department of Orthopedic Surgery, Shanghai Jiao Tong University Affiliated Sixth People's Hospital (Shanghai, China), and were approved by the Ethics Committee of Shanghai Jiao Tong University Affiliated Sixth People's Hospital.

Human umbilical vein endothelial cells (HUVECs) were bought from Keygen Biotech company and cultured in ECM medium with 5% FBS. Human foreskin fibroblast cells (HFF-1) were obtained from Institute of Biochemistry and Cell Biology, Shanghai, and cultured in DMEM medium with 15% FBS.

### 2.2. Diabetic Rat Model Establishment and Tissues Taken

Diabetic rats were obtained according to our previous study [12]. A total of 20 clean male Sprague-Dawley (SD) rats, weighing 250-300 g, were provided by the Experimental Animal Center of the Shanghai Jiao Tong University Affiliated Sixth People's Hospital and were fed as described in reference [12]. 10 rats were selected randomly as the diabetes model group (DM group) and followed by a Streptozotocin (STZ, Aladdin, Shanghai) injection with a dose of 5 mL/kg through the abdominal cavity. STZ was dissolved in citrate-sodium citrate buffer to prepare the STZ solution with a concentration of 10 mg/ml. The rats of the DM group were fed with a high-sugar and high-fat diet for a week. When blood glucose value of rat was ≥16.7 mmol/L, a successful diabetic model was obtained.

After successful anesthesia, the back hair of rats was shaved, and a piece of 2 cm round whole-layer skin was removed from both sides of the back, respectively. The tissue was washed by saline solution wash and stored in liquid nitrogen.

### 2.3. Immunohistochemistry Assay

Primary tumor tissues were fixed with 10% formalin, embedded in paraffin, and cut into slices with 4 *μ*m thickness. The following procedure was performed as previous description [21]. Briefly, after the rats were sacrificed, the wounds were harvested with the surrounding tissue. The tissue specimens were fixed with 4 wt% paraformaldehyde in PBS at 4°C for 24 h and embedded in paraffin to prepare histological sections. The 4 *μ*m thick sections were stained with hematoxylin and eosin. Using a light microscope, specimen was observed and measured.

### 2.4. Immunofluorescence Assay

Tissue processing and blocking were generally the same as the previous immunohistochemistry processing methods. After blocking, tissues were incubated with primary antibody against *α*-SMA, CD31 at 4°C overnight. The next day, the samples were washed three times with PBS, 5 minutes each time. Incubated the tissues with secondary antibodies at room temperature for 50 minutes and staining nuclei with DAPI for 10 minutes. Finally, sealed slides with antifluorescence quenching mounting medium and photographed by a confocal fluorescence microscope (Carl Zeiss, Jena, Germany). The fluorescence intensity was calculated using ImageJ software.

### 2.5. Total RNA Extraction and qRT-PCR

Rapid grinding with liquid nitrogen of the tissue samples was indispensable before total RNA extraction. Total RNA of tissue samples and cells was isolated with TransZol Up (TransGen Biotech, Beijing, China). The PrimeScript™ 1st Strand cDNA Synthesis Kit (TaKaRa, Dalian, China) and the PrimeScript RT Master Mix Perfect Real-Time Kit (TaKaRa, Dalian, China) were used to construct the cDNA library of mRNAs and miRNAs. The expression levels mRNAs or miRNAs were assessed by qRT-PCR using SYBR GreenII (TaKaRa) and a CFX96™ Real-time System (Bio-Rad). 18S rRNA and U6 snRNA were used as the endogenous controls for mRNAs and miRNAs, respectively. The results were processed by the relative quantification (2^-*ΔΔ*Ct^) method for relative quantification of mRNAs and miRNAs. All of primer sequences are shown in Supplementary Table S1.

### 2.6. Cell Transfection

HUVECs and HFF-1 cells were transiently transfected with 50 nM miR-199a mimic/inhibitor, 100 nM VEGFA siRNA (siVEGFA), ROCK1 siRNA (siROCK1), or negative control (siNC) (RIBOBIO, Guangzhou, China) using Invitrogen™ Lipofectamine 2000 (Life Technologies, New York, USA) according to the manufacturer's instructions. After 24 h to 72 h posttransfection, cells were used for qRT-PCR, cell proliferation analysis, wound healing analysis, transwell analysis, and western blot.

### 2.7. Cell Proliferation Assay

The proliferation rates of HUVEC and HFF-1 cells were determined by CCK-8 assay (Cell Counting Kit-8 assay kit, Dojindo, Tokyo, Japan). Briefly, the cells were plated in 96-well plates (Corning) at the density of 2,000 cells/well and incubated at 37°C in a 5% CO_2_ humidified environment. The cells were counted and equally seeded. After the transfection of miR-199a-5p mimic or inhibitor for 24 h, CCK-8 was added and incubated for 2.5 h, followed by the absorbance detection of cells at 24 h, 48 h, and 72 h, respectively.

### 2.8. Cell Migration Assay

The migration rates of cells were determined by the wound healing assay and transwell assay. As for the wound healing assay, cells were plated into 12-well plates (Corning) at a density of 2.5 × 10^5^ cells per well, and continuous culture occurs until the cell density reaches above 90%. The sterile pipette 200 *μ*L tips were used to produce the scratch wounds, and the cells were washed 2-3 times to discard the cell debris by PBS. After incubating with serum-free medium for 8 h (HFF-1) or 24 h (HUVEC), the distances between the wounds were assessed and photographed. Finally, the wound area was determined by Image-Pro Plus software 5.1 (Media Cybernetics, Inc. Siler Spring, MD) in order to perform for quantitative assessment. Migration ratio = (width of wound in 0 h − width of wound in 24 h)/width of wound in 0 h × 100%.

Cell transwell assay was performed by 24-well plates with single chambers, 8000 cells incubated in 100 *μ*L fetal bovine serum-free medium were plated into the upper chamber, and 500 *μ*L medium with 10% fetal bovine serum was added to the lower part of the chamber. After a 24 h migration, the cells were fixed by methanol, stained by crystal violet, and photographed by a phase-contrast inverted microscope.

### 2.9. Western Blot Analysis

To isolate the proteins, cellular total proteins were lysed with RIPA lysis buffer (CWBIO, Beijing, China) and using a Protein BCA Assay Kit (Bio-Rad, Hercules, California, USA) to quantify content of protein. Protein samples were separated by SDS-PAGE and transferred to a polyvinylidene difluoride (PVDF) membrane (Millipore Corporation, Billerica, MA, USA). After blocking in 5% powdered milk for at least 1 h at room temperature, the membranes were incubated by using rabbit anti-ROCK1 and anti-VEGFA antibodies (1 : 1000, Cell Signaling Technology, Danvers, MA, USA) at 4°C overnight. Afterward, washing and incubating the membranes with a horseradish peroxidase- (HRP-) conjugated secondary antibody (1 : 10000, Cell Signaling Technology, Danvers, MA, USA) for 1 h at room temperature. Subsequent visualization was detected using a chemiluminescent HRP substrate (Millipore Corporation, Billerica, USA) and imaged with an E-Gel Imager. Protein levels were normalized to GADPH.

### 2.10. Statistical Analyses

Image analysis was performed using ImageJ software through area statistics with ROI. SPSS v22.0 software was used to analyze data. The results are expressed as the mean ± S.E.M. After verifying that it conformed to the normal distribution, the comparison of the means between the two sets of data was performed using the unpaired, two-tailed, homogeneous variance Student's *T* test. Differences were considered statistically significant when *P* < 0.05. All experiments were performed in triplicate.

## 3. Results

### 3.1. miR-199a-5p Is Increased in Response to Diabetic Stimuli

**Figure 1:**
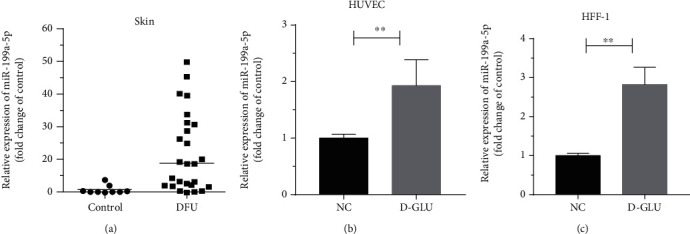
miR-199a-5p is upregulated in DM tissues and high glucose cell model. (a) Relative expression of miR-199a-5P in skin tissue of control group and DFU group. (b, c) The expression levels of miR-199a-5p in HUVEC and HFF-1 cell lines. ∗*P* < 0.05, ∗∗*P* < 0.01.

The expression levels of miR-199a-5p were investigated in lower limb tissue samples from 26 patients with DM and 9 patients with nondiabetic as control. In order to further verify the effect of diabetic high glucose status on wound healing, vascular endothelial cells (HUVEC) and fibroblasts (HFF-1) were used to study the formation of granulation tissue in wound healing for further experimental exploration. The expression of miR-199a in DFU patients was significantly higher than that of health people (Figure 1(a)). In HUVEC and HFF-1 cell cultured by higher content glucose, the expression of miR-199a-5p was much higher than that of control (Figures 1(b) and 1(c)).

### 3.2. miR-199a-5p Inhibits Proliferation and Migration in ECs and in Fibroblasts

**Figure 2:**
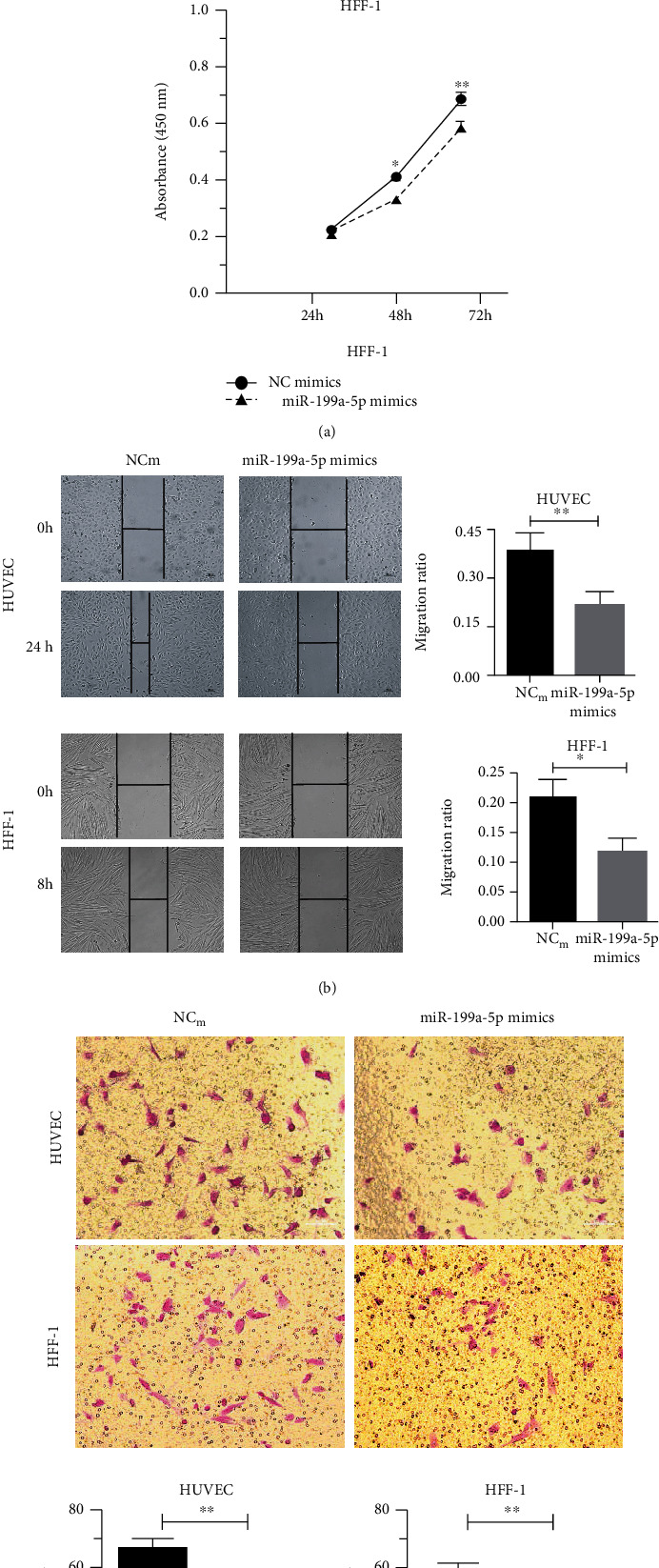
miR-199a-5p could inhibit the proliferation and migration in ECs and fibroblasts. (a) Cell proliferation ability of HUVEC and HFF-1 cells transiently transfected with miR-199a-5p mimic measured by CCK-8 assay for 24 h, 48 h, and 72 h. (b) HUVEC and HFF-1 cells transfected with miR-199a-5p mimic or NC were subjected to wound healing assay and images were taken at 0 h and 24 h. (c) Transwell migration assay performed after transfection of HUVEC and HFF-1 cells with miR-199a-5p mimic or NC for 24 h and 8 h, respectively. The migrated cells were stained with crystal violet and photographed. Migrated cells were counted and analyzed. Each assay was performed in triplicate. ∗*P* < 0.05, ∗∗*P* < 0.01, and ∗∗∗*P* < 0.001.

miR-199a-5p mimic was used to investigate the cellular function of miR-199a-5p in ECs and fibroblasts. First, the cell proliferation was significantly restrained after being transfected with the miRNA mimic by cell counting kit 8 (CCK-8) assays in HUVEC and HFF-1 cells (Figure 2(a)). Then, the effect of miR-199-5p on the migration of HUVEC and HFF-1 cells was verified with wound healing and transwell assays. The results indicated that miR-199a-5p mimic transfected cells migrated toward the wound at a much slower rate than the NC group cells in the wound healing assay (Figure 2(b)) and could reduce the migration of HUVEC and HFF-1 cells in the transwell assay (Figure 2(c)). At the same time, the miR-199a-5p inhibitors were transfected into the cells to downregulate the expression of miR-199a-5p and to further verify the effects of miR-199a-5p on the function of HUVEC and HFF-1 cells. The expression levels of miR-199a-5p and its targets, VEGFA and ROCK1, after miR-199a-5p inhibitor transfection were validated by qRT-PCR and Western blot (Supplementary Figure S1A-C). The cell function of HUVEC and HFF-1 induced by miR-199a-5p overexpression was rescued by miR-199a-5p inhibitor transfection (Supplementary Figure S1D-F).

### 3.3. miR-199a-5p Affects Diabetic Wound Healing via *VEGFA* and *ROCK1*

**Figure 3:**
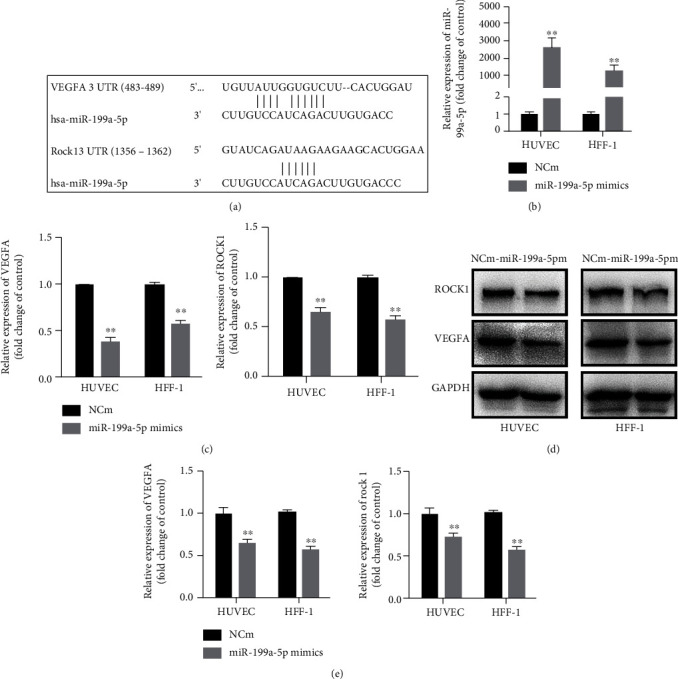
VEGFA and ROCK1 were directly target of miR-199a-5p. (a) Predicted target of miR-199a-5p in the 3′-UTR of VEGFA and ROCK1-mRNA. (b) The relative expression of miR-199a-5p mRNA after upregulating with miR-199a-5p mimic as measured by qRT-PCR. (c) The expression mRNA levels of VEGFA and ROCK1 in HUVEC and HFF-1 cell lines after transfected with miR-199a-5p mimic or NC mimic. (d) Protein levels of VEGFA and ROCK1 in HUVEC and HFF-1 cell lines after transfected with miR-199a-5p mimic or NC mimic. ∗*P* < 0.05, ∗∗*P* < 0.01, and ∗∗∗*P* < 0.001.

As previously reported, miR-199a-5p can simultaneously target VEGFA and ROCK1 [22–24], which have corresponding targeted binding sites (Figure 3(a)). In order to explore whether the roles of miR-199a-5p on diabetic wound healing were mediated by VEGFA and ROCK1, we detected the expression levels of VEGFA and ROCK1 in miR-199a-5p overexpression cells. The transfection effects of miR-199a-5p in HUVEC and HFF-1 cells were confirmed by qRT-PCR (Figure 3(b)). As expected, the mRNA and protein levels of VEGFA were decreased by miR-199a-5p in HUVEC and HFF-1 cells significantly through qRT-PCR and western blotting detection (Figures 3(c)–3(e)).

### 3.4. Silencing VEGFA and ROCK1 Inhibits Proliferation and Migration of ECs and Fibroblasts

**Figure 4:**
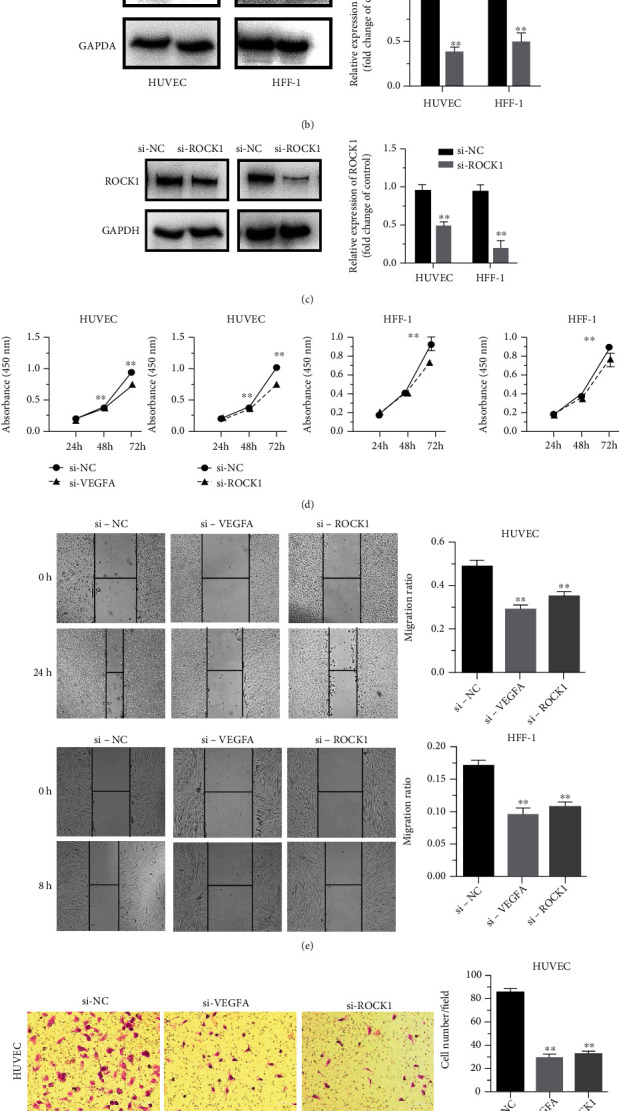
Knockdown of VEGFA and ROCK1 reduces cell proliferation and migration of ECs and fibroblasts. (a)–(d) The levels of VEGFA and ROCK1 mRNA and protein in HUVEC 24 h and HFF-1 8 h cells transfected with siRNA (si-VEGFA, si-ROCK1) were measured by qRT-PCR and western blot, respectively. (e) Proliferation of HUVEC and HFF-1 cells transfected with si-VEGFA/si-ROCK1 or NC was determined by CCK-8, which was tested in 24 h, 48 h, and 72 h. (f) HUVEC and HFF-1 cells transfected with si-VEGFA/si-ROCK1 or NC were subjected to wound healing assay and images were taken at 0 h and 24 h. (g) Transwell migration assay performed after transfection of HUVEC and HFF-1 cells with si-VEGFA/si-ROCK1 or NC. The migrated cells were stained with crystal violet and photographed. ∗∗*P* < 0.01 and ∗∗∗*P* < 0.001.

To validate the regulatory role of VEGFA and ROCK1 in ECs and fibroblasts, VEGFA and ROCK1 were silenced by using siRNA. Our results revealed that the expression levels of VEGFA and ROCK1 mRNA (Figure 4(a)) and protein (Figures 4(b) and 4(c)) were significantly downregulated in HUVEC and HFF-1 cells, which were transfected with siVEGFA and siROCK1, respectively, compared with the siNC. Furthermore, cell proliferation showed that transfect with siVEGFA and siROCK1 has a significant suppression in HUVEC and HFF-1 cells, compared with that of the siNC transfected cells (Figure 4(d)). Then, the effect of siVEGFA and siROCK1 on the migration of HUVEC and HFF-1 cells was done with wound healing and transwell assays. The results indicated that siVEGFA and siROCK1 could inhibit the migration of HUVEC and HFF-1 cells in the transwell assay, and it migrated toward the wound at a much slower rate than the NC group cells in the wound healing assay and transwell (Figures 4(e) and 4(f)).

### 3.5. Downregulating miR-199a-5p Accelerates Cutaneous Wound Healing in a Diabetic Rat Model

**Figure 5:**
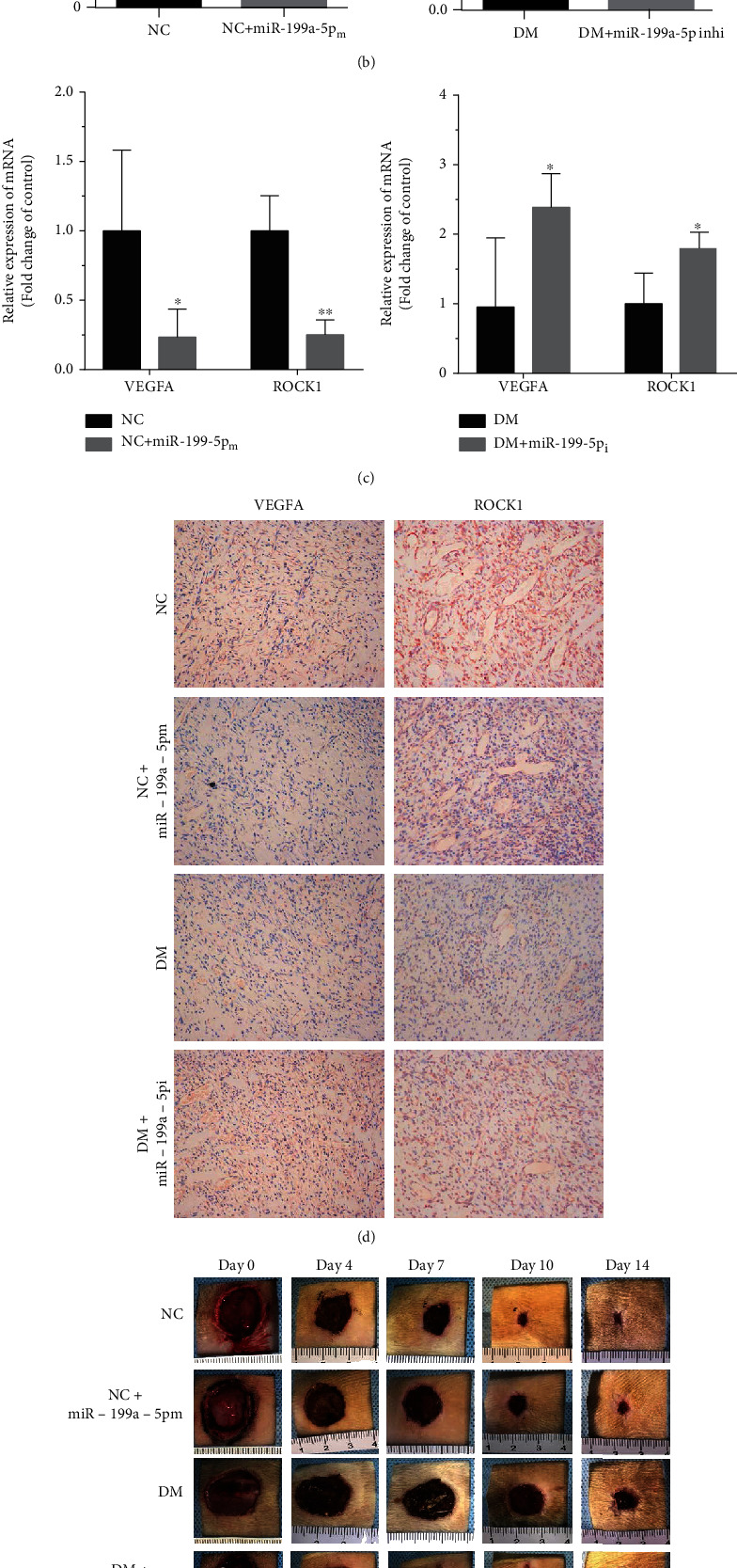
Downregulating miR-199a-5p accelerates cutaneous and improves granulation tissue formation wound healing in a diabetic rat model. (a) Representative images of full-thickness skin defects in rats of NC group, NC + miR − 199a − 5pm group, DM group, and DM + miR − 199a − 5pi group immediately, 4, 7, 10, and 14 days postoperatively. (b) Wound closure rate (%). (c) Representative images of (50×, scale bar 200 *μ*m) H&E stained sections of the NC group, NC + miR − 199a − 5pm group, DM group, and DM + miR − 199a − 5pi group 7 and 14 days postoperatively. The weights tumors isolated from mice after 7 weeks. (d) Illustration of measuring granulation tissue thickness (50×, scale bar 200 *μ*m). (e) Granulation tissue thickness 7 and 14 days postoperatively (*μ*m). ∗*P* < 0.05, ∗∗*P* < 0.01, and ∗∗∗*P* < 0.001.

We used the models of diabetic on rat to explore the treatment effect of miR-199a-5p *in vivo*. For the wounds were injected with miR-199a-5p agomiR/antagomiR, compared with the NC-treated group in normal and DM rat, the therapeutic effect of miR-199a-5p about diabetic rat picture began to show in 0 day, 4 day, 7 day, 10 day, and 14 day. We can observe that the wound at five times nodes, in the NC group, NC + miR − 199a − 5p agomiR group, DM group, and DM + miR − 199a − 5p antagomiR group, the wound area decreased with pass of time (Figure 5(a)). In general, the healing rate of nondiabetic rats is faster than that of diabetic rats. Although the healing rate of the two groups of nondiabetic rats (NC group and NC + miR − 199a − 5p agomiR group) is statistically different, the wound healed almost completely between 10 and 14 days after surgery (Figure 5(b)). However, rats in the diabetic group (DM group and DM + miR − 199a − 5p antagomiR group) did not heal completely on the 14th day (Figure 5(c)).

Next, we observed the rat full-thickness skin defect model by H&E staining. The observation site is the junction of the wound and normal skin tissue to ensure that the observation site is the new granulation tissue and the new skin tissue. The results showed that diabetic rats (DM group and DM + miR − 199a − 5p antagomiR group) had worse healing compared with nondiabetic rats (NC group and NC + miR − 199a − 5p agomiR group) (Figure 5(d)). After 7 days of operation, H&E staining results showed that the inflammatory cells in each group were infiltrated obviously, the epidermal layer was hyperplasia and thickened, and the new granulation tissue and epithelial tissue migrated from the wound edge to the wound center. After 14 days of operation, the wound tissues in the nondiabetic rats (NC group and NC + miR − 199a − 5p agomiR group) were almost completely covered by epithelial cells, but the new skin tissue in the NC + miR − 199a − 5p agomiR group was disordered, with more inflammatory cell infiltration and less sebaceous gland structure compared with NC group. Nevertheless, the wound in the diabetic rats (DM group and DM + miR − 199a − 5p antagomiR group) did not heal, the epithelial tissue did not completely cover the wound, the inflammatory reaction was severe, the skin was thin, and the normal arrangement and accessory structures were lacking on the 14th day (Figure 5(d)). Thus, downregulating miR-199a-5p can improve the quality of wound healing.

Then, the thickness of granulation tissue in nondiabetic rats was greater than that in diabetic rats on the 7th and 14th day after operation (Figures 5(e) and 5(f)). It was concluded that downregulating miR-199a-5p could promote the healing rate of diabetic wound and improve granulation tissue formation in DM.

### 3.6. miR-199a-5p Promotes Angiogenesis in the Cutaneous Wound Areas of Diabetic Rats

**Figure 6:**
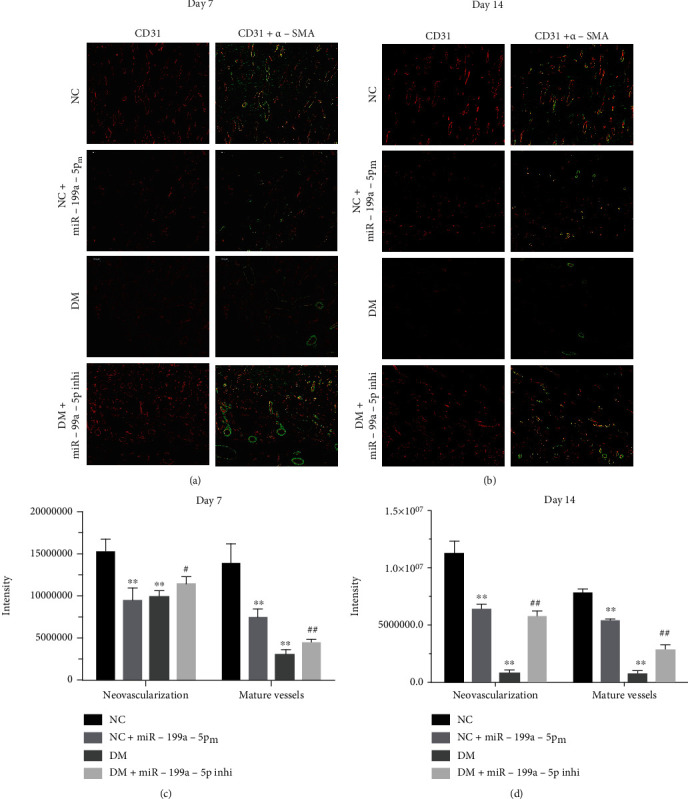
miR-199a-5p promotes angiogenesis in the cutaneous wound areas of diabetic rats. Representative images (100×, scale bar 100 *μ*m) of immunofluorescent staining for CD31 (red) and *α*-SMA (green) of NC group, NC + miR − 199a − 5pm group, DM group, and DM + miR − 199a − 5pi group. Newly formed blood vessels were defined by positive CD31 staining. Mature blood vessels were defined by positive CD31 and *α*-SMA staining. Representative images of immunofluorescent staining for CD31 (red) and *α*-SMA (green) (a) 7 days postoperatively and (b) 14 days postoperatively. Number of newly formed blood vessels and mature blood vessels (c) 7 days postoperatively and (d) 14 days postoperatively. ∗∗*P* < 0.01 compared to NC group, ^#^*P* < 0.05 compared to DM group, ^##^*P* < 0.01 compared to DM group.

To assess the effect on angiogenesis by miR-199a-5p, immunofluorescent (IF) was used in this experiment. Newly formed blood vessels were defined by positive CD31 staining. Mature blood vessels were defined by positive CD31 and *α*-SMA staining. IF staining for CD31 (red) and *α*-SMA (green) 7 days postoperatively and 14 days was performed postoperatively (Figures 6(a) and 6(b)). In nondiabetic rats, the intensity of new blood vessels and mature blood vessels of the NC + miR − 199a − 5p_m_ group was significantly lower than that of the NC group in 7 and 14 days after operation, separately (Figure 6(c)). In diabetic rats, the intensity of new blood vessels and mature blood vessels in the DM + miR − 199a − 5p_i_ group was significantly higher than that in the DM group post 7- and 14-day operation (Figure 6(d)).

## 4. Discussion

miRNAs are involved in the development of a variety of cancers and chronic diseases. Usually, miRNAs function as promoters or inhibitors in the progress of disease [25]. Studies have found that miR-411 can promote lung cancer progression [26]. miR-34a inhibits progression of lung cancer via targeting EGFR, a cancer-drive gene [27]. miR-199a-5p was associated with a poor prognostic phenotype and inhibited proliferation and metabolism by targeting in colorectal cancer [22]. miR-199a also influenced cell angiogenesis, which was detected by tube formation assay. Ghosh et al. found that miR-199a-3p inhibited angiogenesis through targeting VEGFA, VEGFR1, VEGFR2, HGF, and MMP2 in hepatocellular carcinoma [28]. Wang et al. also found that miR-199a-3p inhibited angiogenesis by targeting the VEGF/PI3K/AKT signaling pathway in an in vitro model of diabetic retinopathy [29].

Yang et al. reported that miR-199a-5p was sponged to hsa_circ_0060450, releasing target gene SHP2, and showed it suppressed the JAK-STAT signaling pathway triggered by type I interferon (IFN-I) to inhibit macrophage-mediated inflammation in T1DM [30]. Lin et al. found that miR-199a-5p was upregulated in pancreatic *β*-cells in response to high glucose and promotes apoptosis and ROS generation by targeting SIRT1 in T2DM [31]. And Wang et al. also investigated miR-199a-3p role in DM [Wang H, Wang Z, Tang Q]. Reduced expression of microRNA-199a-3p is associated with vascular endothelial cell injury induced by type 2 diabetes mellitus [32]. They found miR-199a-3p expression was reduced in patients with T2DM compared with healthy subjects. It suggested miR-199a-3p may function as miR-199a-5p in our research.

In the pathogenesis of diabetic wounds, VEGFA is activated in foot skin [33, 34]. This will impair the balance of ECM synthesis and degradation and result in unhealed wounds. In this study, we explored the relationship between VEGFA and ROCK levels and the severity of DFUs.

Then, we focused on VEGFA and ROCK posttranslational regulation. Our research before found that some miRNAs can regulate NF-*κ*B signaling and affect inflammation [35, 36], and it means noncoding RNAs will play more important role in DFU. Noncoding RNAs, such as long noncoding RNA [37, 38], circRNA [39–41], and microRNA [41, 42], play pivotal roles in wound healing. Here, we analyzed miRNA-199a-5p expression in the samples of patients with DFUs.

Compared with the normal wounds, the inflammatory period of diabetic foot ulcer wounds is abnormally prolonged, which makes it difficult to heal the ulcer wound and is easy to recur [43, 44]. miRNAs also play pivotal roles among the inflammation stages of DFU [45, 46]. In this study, HE staining of histological sections showed that compared with the DM + miR − 199a − 5pi group, severe infiltration of inflammatory cells in the wound granulation tissue was detected in the DM group, which suggested aggravated effects of miR-199a-5p on the inflammatory reaction of DFU.

**Figure 7:**
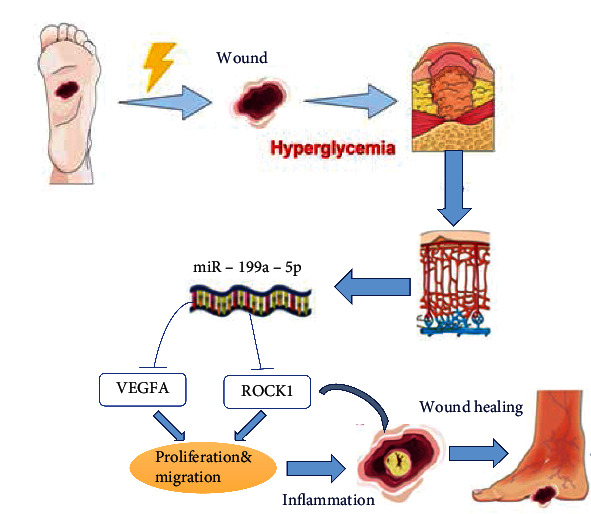
Possible mechanism of miR-199a-5p and VEGFA (ROCK1) axis in DM.

miRNA mimics or inhibitors have been confirmed to be potential drug for nonhealing wounds [47, 48]. In this study, subcutaneous injection of miR-199a-5p agomir accelerated diabetic wound healing, improved the skin thickness in a diabetic wound animal model through decreased VEGFA and rock the protein expression level, increased collagen content, and enhanced migration of keratinocytes. As mentioned above, *in vitro* experiments showed that the overexpression or inhibition of miR-199a-5p resulted in the downregulation or upregulation of VEGFA and ROCK expression, respectively, and the concomitant change of VEGFA and ROCK protein levels in EC and HIFF cells. Furthermore, miR-199a-5p antagomiR also showed an outstanding healing effect for the wound injury caused by inflammation *in vivo* (Figure 7).

## 5. Conclusion

In summary, our findings demonstrate an important role for miR-199a in diabetic wound healing. We found that the expression of miR-199a-5p was significantly increased in the skin tissues of DFU samples, meanwhile, VEGFA and ROCK1 were direct targets of miR-199a-5p. Overexpression of miR-199a-5p arrested the cell proliferation, migration, and invasion of HUVEC and HFF-1 cells through the inhibition of VEGFA and ROCK1. *In vivo*, inhibition of miR-199a-5p promoted the wound healing rate and angiogenesis in the cutaneous wound areas of diabetic rats. Accordingly, these findings give insight into miR-199a-5p potential use and therapeutic targets to reduce complications from diabetic wounds.

## Data Availability

Data are available upon request.

## Supplementary Materials

Supplementary Materials Inhibition of miR-199a-5p rescues impaired proliferation and migration in ECs and in fibroblasts. (Figure Supplementary 1). Supplementary Table S1.
