# Supplementary material for: miR-199a-5p Plays a Pivotal Role on Wound Healing via Suppressing VEGFA and ROCK1 in Diabetic Ulcer Foot
Source: Oxid Med Cell Longev. 2022 Apr 7;2022:4791059. doi: 10.1155/2022/4791059 (PMC9010206; doi:10.1155/2022/4791059)
Supplement: Supplementary Materials — Inhibition of miR-199a-5p rescues impaired proliferation and migration in ECs and in fibroblasts. (Figure Supplementary 1). Supplementary Table S1. [file 4791059.f1.docx]

**Inhibition of miR-199a-5p rescues impaired proliferation and migration in ECs and in fibroblasts. (Figure Supplementary 1)**

**
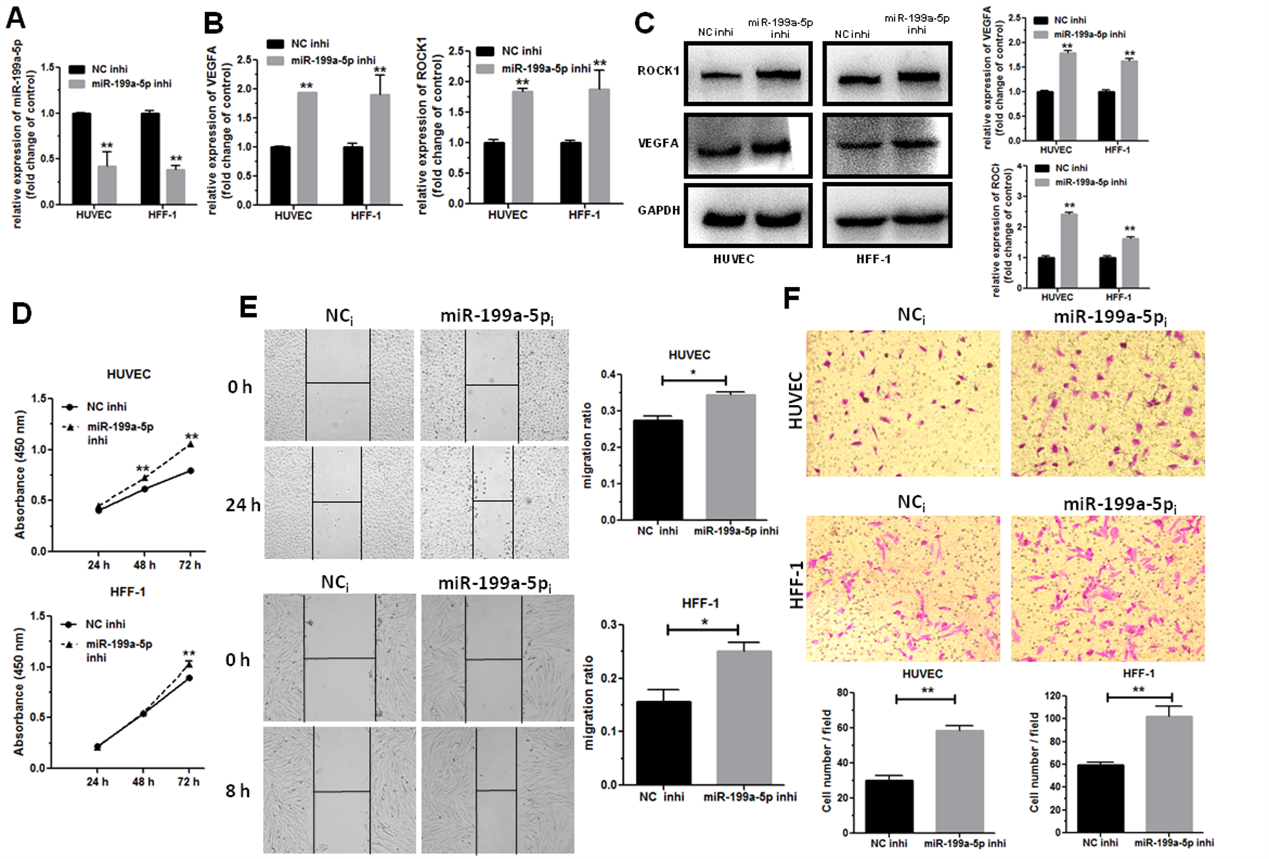
**

**Inhibition of miR-199a-5p rescues impaired proliferation and migration in ECs and in fibroblasts.** (A) Downregulation of miR-199a-5p following transfected with miR-199a-5p inhibitor in HUVEC and HFF-1cells. (B-C) The levels of VEGFA and ROCK1 mRNA and protein in HUVEC 24 h and HFF-1 8 h cells transfected with miR-199a-5p inhibitor or NC were measured by qRT-PCR and western blot, respectively. (D) Inhibition of miR-199a-5p rescued impaired cell proliferation in both HUVEC and HFF-1 cell lines in the context of diabetic stimuli, which tested in 24h, 48h, 72h. (E) Representative photographs showing the migration of HUVEC and HFF-1 cells transfected with miR-199a-5p inhibitor or NC groups in scratch assay, and quantitation cell count, after 24h in HUVEC and 8h in HFF-1 cell. (F) Representative photographs showing the migration of HUVEC and HFF-1 cells of miR-199a-5p inhibitor or NC groups in transwell assay, and quantitation cell count. *P<0.05, **P<0.01, and ***P<0.001.

**Supplementary Table S1**

The sequence of the primers used in this study

| Primer | Sequence（5'-3'） |
| --- | --- |
| 18S RNA(F) | AGGAATTCCCAGTAAGTGCG |
| 18S RNA(R) | GCCTCACTAAACCATCCAA |
| U6 snRNA(F) | CTCGCTTCGGCAGCACA |
| U6 snRNA(R) | AACGCTTCACGAATTTGCGT |
| VEGFA qRT-PCR(F)  VEGFA qRT-PCR(R) | CGCAAGAAATCCCGTCCCTG  TTAACTCAAGCTGCCTCGCC |
| ROCK1 qRT-PCR(F) | TTGGTTGAAATTGCTTTCCGCT |
| ROCK1 qRT-PCR(R) | AAAGCATCCAATCCATCCAGC |
| miR-199a-5p | CCCAGUGUUCAGACUACCUGUUC |
